# Study on the effect of blood flow restriction training combined with IASTAM on ankle strength and function intervention in athletes with chronic ankle instability in sport dance events

**DOI:** 10.1186/s13102-024-00873-x

**Published:** 2024-04-11

**Authors:** Yang Liu, Ying Wang

**Affiliations:** Graduate School, School of Arts, Wuhan Sports University, 430079 Wuhan, China

**Keywords:** Chronic ankle instability, Ankle sprain, Sports dance, Blood flow restriction training, Ankle stability training, Balance training, Instrument-assisted soft tissue mobilization

## Abstract

**Background:**

Athletes engaged in sports dance frequently encounter the potential for ankle injuries and instability, factors that may contribute to diminished training efficacy, compromised athletic performance, prolonged recuperation, and heightened susceptibility to recurring injuries.

**Objective:**

The objective of this study was to investigate the impact of an exercise intervention (comprising blood flow restriction training combined with low-load ankle muscle strength training and balance training) as well as instrument-assisted soft tissue mobilization (IASTM) on the foot and ankle function, strength, and range of motion in sports dance athletes exhibiting ankle instability (CAI).

**Methods:**

Thirty participants exhibiting ankle instability, restriction, or discomfort were recruited and randomly assigned to two groups: the Test group (comprising blood flow restriction training combined with IASTM, *n* = 15) and the traditional ankle strength training group (*n* = 15). The intervention spanned 4 weeks, with one session per week. Assessment of the Cumberland Ankle Instability Tool (CAIT), Foot and Ankle Ability Measure (FAAM), and ankle range of motion occurred at three time points: pre-intervention, immediately following the initial intervention, and after 4 weeks of intervention. Ankle strength testing was conducted solely before and after the intervention for comparative analysis.

**Results:**

There were no significant variances in baseline characteristics between the two intervention groups. In terms of CAIT scores, both groups exhibited notably higher scores following the initial intervention and after 4 weeks of intervention compared to pre-intervention (*P* < 0.05). The Test group displayed higher CAIT scores than the control group, signifying a more pronounced enhancement in ankle stability among patients in the Test group. Concerning FAAM scores, both groups significantly enhanced ankle function in CAI patients (*P* < 0.05), with the Test group demonstrating notably higher FAAM-SPORT scores than the control group (*P* < 0.05), indicating superior restoration of athletic capability in the Test group. As for improvements in ankle range of motion, both groups demonstrated significant enhancements compared to pre-intervention (*P* < 0.05). The Test group exhibited significantly superior improvements in dorsiflexion, eversion, and inversion range of motion compared to the control group (*P* < 0.05), while the control group did not exhibit significant enhancements in plantarflexion and eversion range of motion (*P* > 0.05). Both groups displayed enhanced ankle strength in CAI patients following the intervention (*P* < 0.05), with the Test group manifesting notably higher dorsiflexion and inversion strength than the control group (*P* < 0.05).

**Conclusion:**

Both blood flow restriction training combined with IASTM and traditional ankle strength and stability training have shown significant improvements in stability, function, strength, and range of motion in CAI patients. Furthermore, the Test group exhibits superior efficacy in ankle stability, daily functional movement, dorsiflexion, and eversion range of motion compared to the control group.

**Clinical trial registration:**

9 February 2024, ClinicalTrials.gov, ID; NCT06251414.

## Introduction

Introduction Sports dance, also known as international standard dance, encompasses two series: Latin and Modern. In this high-intensity sport, athletes frequently encounter various risks of sports injuries, with Chronic Ankle Instability (CAI) being a prevalent and significant concern in sports dance [1, 2]. Repetitive sprains and prolonged abnormal ankle joint function during physical activity are pivotal factors contributing to CAI [3, 4]. The primary hallmark of CAI is diminished ankle stability, heightening the likelihood of recurring ankle sprains during walking, sports, or other activities [4, 5]. For sports dance athletes, the foot and ankle joints endure substantial stress during movement. The intricate structure and frequent movement demands render the ankle joint more susceptible to injury. Ankle injuries exhibit a strong correlation with the nature of sports dance, as dancers are required to execute high-frequency and high-difficulty movements, subjecting the ankle joint to dynamically changing loads. This movement pattern exposes the ankle to a heightened risk of injury [6]. Particularly in dance styles necessitating frequent changes in direction and jumping movements, ankle sprains and ligament strains are prevalent forms of injury [7]. Moreover, apart from the unpredictable stresses imposed on the ankle during dance movements, continuous high-intensity dance training and performances can elevate the risk of ankle muscle fatigue and subsequent injuries [8]. Ankle joint injuries not only impact athletes’ athletic performance but also lead to setbacks in the rehabilitation process, adversely affecting their professional careers. Following the resolution of symptoms subsequent to a period of rest, the muscles surrounding the ankle may remain weakened due to trauma or inadequate rehabilitation, encompassing muscles around the foot and ankle such as the gastrocnemius and tibialis anterior. Muscle weakness or incomplete ligament healing can result in inadequate joint support, culminating in a lack of ankle stability, which is also a pivotal factor contributing to CAI [9, 10]. Additionally, neural control over muscle movement may be compromised, leading to insufficient muscle coordination and an increased risk of ankle joint instability [11, 12]. Consequently, post-rehabilitation strengthening exercises targeting ankle strength and stability assume particular significance.

Blood Flow Restriction Training (BFRT) is a specialized training technique designed to stimulate muscle growth and enhance strength by employing cuffs or elastic bands to restrict blood flow in the limbs [13]. Initially developed for rehabilitating injured athletes, BFRT can augment muscle strength under reduced loads, aiding in the prevention of further injuries during the recovery process [14]. As research has progressed, BFRT has gradually found application across a wider spectrum of fitness and training domains [15, 16]. A key advantage of BFRT lies in its capacity to facilitate effective training at relatively light loads, thereby reducing stress on joints and tendons, rendering it suitable for individuals who may struggle with high-intensity training due to injury or other factors [14]. Furthermore, BFRT has the potential to yield muscle growth and strength gains in a shorter timeframe, rendering it a time-efficient training method [14]. BFRT can be utilized not only for strength training but also for rehabilitation, endurance enhancement, and the improvement of athletic performance, making it versatile across various disciplines [15]. In the realm of rehabilitation training, it can be employed on multiple body parts to expedite recovery and bolster muscle strength in the affected area, exerting direct effects on limb muscles and elbow and knee joints, as well as indirect effects on the shoulder, hip, and gluteal regions [17–20]. Research indicates that BFRT elicits an activating effect on the calf muscle group in CAI athletes, leading to a notable decrease in calf muscle oxygen saturation and a significant increase in muscle fatigue perception scores during low-load resistance exercise, thereby fostering lower limb strength and function in CAI patients [21–23].

Instrument-Assisted Soft Tissue Mobilization (IASTM) is a physical therapy technique that employs specially designed tools, such as metal or plastic scraping boards, to complement manual therapy in addressing soft tissue issues [24]. It is primarily utilized in rehabilitation medicine, sports medicine, and orthopedic surgery to alleviate tension, adhesions, pain, and movement dysfunction in muscles, fascia, and tendons [25–27]. The edge design of IASTM tools facilitates the release of adhesions in tissues, thereby enhancing tissue elasticity and plasticity. This modulation of the pathological area through neural pathways serves to alleviate pain and improve nerve function [28]. Various types of treatment tools enhance treatment precision, bolster blood circulation, expedite the recovery process, improve tissue elasticity, and expand joint range of motion, all with lower risks and complications compared to invasive surgery [29–31]. Research has demonstrated that IASTM can significantly enhance lower limb joint function, diminish pain, and increase range of motion [19, 28].

Recent studies have indicated that the combination of IASTM with BFRT yields a significant reduction in patellofemoral joint pain, an improvement in knee soft tissue flexibility, and enhanced lower limb muscle strength and function in patients. In terms of overall therapeutic effects, the combined treatment surpasses sole IASTM therapy, underscoring the efficacy of the integrated approach [19]. Therefore, to further optimize the rehabilitation process for CAI, this study has devised a comprehensive rehabilitation training program that integrates Blood Flow Restriction Training (BFRT) with ankle balance training, ankle strengthening exercises, and IASTM physical mobilization. BFRT was selected due to its capacity to induce muscle strength adaptations at lower loads, offering a relatively low-risk approach to rehabilitation. Through the amalgamation of ankle balance training, strengthening exercises, and physical mobilization via IASTM, a synergistic effect is anticipated to enhance the rehabilitation process. By comparing conventional balance training with pure BFRT training, this study aims to assess the impact of BFRT-assisted rehabilitation on ankle recovery in sports dance athletes, providing novel theoretical and empirical support in the realm of rehabilitation. Through comprehensive research, the aim is to furnish more effective and holistic rehabilitation programs for sports dance athletes, thereby enhancing their recovery speed and outcomes, consequently safeguarding their health and professional careers.

## Methods

### Research objects

Students specializing in sports dance at the school who exhibited chronic ankle instability were selected for participation. Subsequently, balance testing was conducted using the Single Leg Stance [32], Trendelenburg test [33], Dynamic Balance Test [34], Anterior Drawer Test [35], and Y-Balance Test [36]. If two or more tests out of these five yielded positive results, indicating chronic ankle instability [5, 37], the individuals were included as subjects in the experiment. The final cohort comprised 30 participants, all of whom had provided informed consent. This study has received approval from the Ethics Review Committee of the Medical School at Wuhan Sports University (Acceptance number: whsu2023102) and has been registered under the identification NCT06251414 on the ClinicalTrials.gov platform. The experiment was conducted in the sports rehabilitation laboratory of our school. Specific inclusion and exclusion criteria for the participants can be found in Table 1.

**Table 1:** Inclusion and exclusion criteria

Criteria	Inclusion Criteria	Exclusion Criteria
Age	18–35 years old	Under 18 or over 35 years of age
Disease duration	Have symptoms of chronic ankle instability for at least 3 months	Acute ankle injury or no joint injury
CAIT score	Have a CAIT score less than or equal to 24	CAIT score higher than 24
Functional Screening	Pass a pre-laboratory ankle function screen with 2 or more positive tests	Failed ankle function screening
Structural Examination	Not have a structural joint lesion or congenital ankle deformity.	Presence of structural ankle pathology or congenital ankle deformity
Medical History	No previous ankle surgery or presence of external injuries	Have undergone ankle surgery or have significant trauma or wounds, Or there may be issues such as skin irritation, infection, open wounds, anemia, hypotension, etc.
Health status	Have no serious cardiac, pulmonary, neurological or other systemic disease.	Have a serious cardiac, pulmonary, neurological, or other systemic condition
Consent to participate in the study	Subjects with sufficient exercise ability to complete a certain intensity and duration of exercise load.	Do not agree to participate in the study or are unable to understand and comply with the study protocol

### Experimental group

This study employed a randomized controlled trial design using simple randomization based on the chronological order of subject recruitment. The initial 15 recruited subjects were assigned to the experimental group. Additionally, the study adopted a single-blind format to ensure that only the experimenters were aware of the specific group assignments, while the subjects themselves remained unaware of their respective group allocations.

**Fig. 1:**
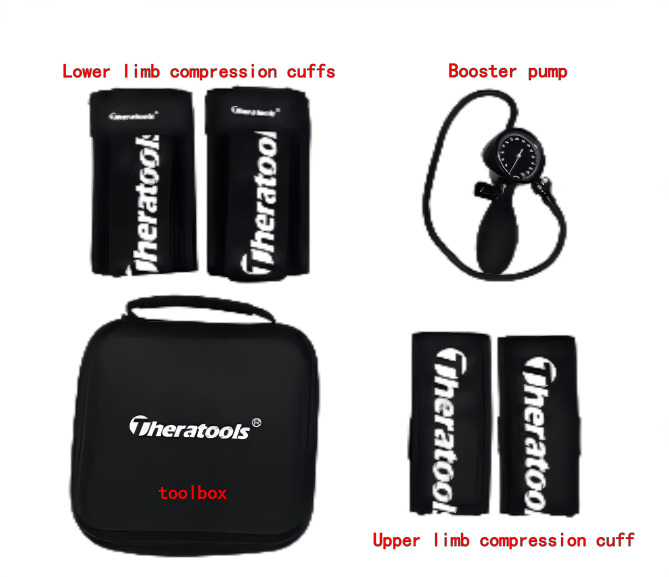
BFRT equipment

The experimental group implemented exercise intervention comprising BFRT combined with IASTM as the primary intervention measures. The BFRT equipment primarily included a pneumatic pump and lower limb occlusive cuffs (refer to Fig. 1), and the applied training protocols encompassed ankle stability exercises and strength training for the muscles around the ankle joint. Ankle stability training involved interventions utilizing the Bosu ball [38], encompassing single-leg support training, kicking balance training, plank support, and squat exercises. Muscle strength training for the ankle joint primarily focused on heel raise exercises and resisted dorsiflexion, eversion, and inversion training using elastic bands. Given the common limitations in dorsiflexion and insufficient strength in eversion among CAI patients, the exercise intervention plan emphasized the intensity of dorsiflexion and eversion training. Specific training protocols can be found in Table 2.

**Table 2:** BFRT ankle training movements

Movement	Steps	Frequency	Training Intensity	Pressurization Value
Heel Lift	1. Subjects wearing BFRT equipment stood at a certain height step (10–15 cm), body upright, knee joints slightly bent toes naturally facing forward, forefoot on the edge of the step, both heels hanging in the air, hands on the wall or chair to maintain body balance. 2. Exhale during the centripetal phase, stretch the ankle joints and fully contract the back of the calves to stand on tiptoe. 3. During the centrifugal phase, inhale, flex the ankle joints, sink the body weight, and maximize the elongation of the back of the calves.	2 times a week / 4 weeks in total	1 set of 12–15 reps / 4–6 sets total	20-50mmHg
Resisted dorsiflexion	1. The subject sits on the floor wearing BFRT equipment with legs extended. The elastic band is placed around the back of the affected forefoot and the subject holds the ends of the band with both hands. 2. During the centripetal phase, the subject exhaled, dorsiflexed the ankle joint, pulled the elastic band to give maximum resistance, and held it at the extreme angle of dorsiflexion for 3–5 s. 3. During the centrifugal phase, inhale, extend the ankle joint, slowly release the tension from the elastic band, and return the ankle joint to its initial position.	2 times a week / 4 weeks in total	1 set of 15–20 reps / 6 sets in total	20-50mmg
Resisted Hallux Valgus	1. The subject sits on the floor wearing BFRT equipment with legs extended. The elastic band is wrapped around the outside of the affected foot, and the experimenter holds both ends of the band with both hands. 2. During the centripetal phase, the subject exhaled, turned the ankle out, pulled the elastic band to give maximum resistance, and held it at the extreme angle of the foot’s turning out for 3–5 s. 3. During the centrifugal phase, inhale, invert the ankle joint, slowly release the tension on the elastic band, and return the ankle joint to its initial position	2 times a week / 4 weeks in total	1 set of 15–20 reps / 6 sets in total	20-50mmg
Resisted Hallux Valgus	1. The subject sits on the floor wearing BFRT equipment with legs extended. The elastic band is placed around the medial aspect of the affected foot. 2. During the centripetal phase, exhale, turn the ankle inward, pull the elastic band to give maximum resistance, and hold it at the extreme angle of foot valgus for 3–5 s. 3. Inhale during the centrifugal phase, turn the ankle out, slowly release the tension on the elastic band and return the ankle to its initial position.	2 times a week / 4 weeks in total	1 set of 10–12 reps / 3 sets total	20-50mmg
Bosu ball single leg support training	1. Place the Bosu ball on the ground with the ball facing upwards and the flat surface facing downwards, the subject wears the BFRT equipment and stands on the ball with one leg. Without the aid of any supporting objects, the subject stands with one leg slightly bent, takes a deep breath, and maintains supported standing for 30–60 s.	2 times a week / 4 weeks in total	1 set of 30–60 s / 3 sets in total	20-50mmg
Bosu Ball Kick Balance	1. Place the Bosu ball on the ground, with the surface of the ball facing upwards and the flat surface facing downwards, and stand on the surface of the ball with one leg while wearing the BFRT equipment. Without the aid of any supporting objects, the subject stood with one leg slightly flexed, and the healthy leg remained flexed at 90° to complete the leg raising movement. 2. Exhale during the centripetal phase and raise the healthy leg to the highest point. During the centrifugal phase, inhale and slowly lower the healthy leg back to the initial position.	2 times a week / 4 weeks in total	1 set of 10–15 s / 3 sets in total	
Bosu Ball Plank Support	Place the Bosu ball on the ground with the flat surface facing up and the ball surface facing down. Subjects wear BFRT equipment and place their legs on the Bosu ball surface to complete the 30–60 surface plank support maneuver.	2 times a week / 4 weeks in total	1 set 30–60 s/total 2 sets	20-50mmg
Bosu Ball Squat	1. Place the Bosu ball on the ground with the flat surface facing up and the ball surface facing down, the subject wears BFRT equipment and places his/her legs on the plane of the Bosu ball and completes the squat with no weight bearing. 2. Exhale during the centripetal phase, the subject extends the hips, stands up slowly, straightens the knee joints, restores the body to its initial position, and keeps the ankle joints stable during the squat. During the centrifugal phase 3. Inhale, the subject flexed the hips and slowly squatted until the knee was below 90° of flexion, keeping the ankle joint stable.	2 times a week / 4 weeks in total	1 set of 15–20 reps / 4–6 sets in total	20-50mmg

In this experiment, IASTM was performed using a fascial blade as the treatment tool. There were five main blade types, including C-shaped - sweeping blade, B-shaped - bat blade, M-shaped - large M blade, A-shaped - shark blade, and S-shaped - hook blade. Each blade had a unique shape and served different purposes accordingly. IASTM intervention was conducted prior to BFRT, with the primary goal of utilizing the physical intervention of the fascial blade to mobilize the soft tissues around the lower leg and ankle joint, aiming to ameliorate ankle joint pain and restore ankle joint range of motion. Specific steps for ankle IASTM procedures can be found in Table 3.

**Table 3:** Steps in IASTM treatment

Knife Type	Operating Method	Strength	Time	Purpose
Type C - Sweeping Knife	With the subject lying prone, a fascial lubricant was evenly applied to the posterior/anterior side of the calf, and the C-probe was used to apply pressure to the target muscle group at a 45° tangential angle in both directions, bottom to top or top to bottom, following the course of the calf muscle fibers.	Low	1 min	Subjects were gradually acclimatized to the rhythm of the instrumental treatment while areas of calf fascia densification or granulation were identified [39–41].
Type A-Shark knife	Slow, repetitive pressure sliding for areas of high resistance in areas of posterior/anterior calf fascial densification or excitatory pain points [42]	Low-Medium	2 min	Soft tissues are loosened in both resting and maximal extension of the calf to restore soft tissue elasticity in areas of stiffness and to reduce or eliminate painful spots.
Type B - Bat Knife	Sliding compressions were performed by applying pressure to the posterior/anterior calf treatment area at an angle of about 45°, in both top-to-bottom and bottom-to-top directions, and small, repeated pressure slides were applied to areas of fascial densification or points of excitation.	Medium-High	3–5 min	Deeper myofascial release of the posterior/anterior calf using highly focused and greater pressure.
M type - Big M knife	Subjects were asked to complete dorsiflexion and toe-flexion movements separately and without interruption, reaching the limit of each movement and holding it for 3–5 s at the maximum angle. Passive fascial knife pressure sliding was performed with breathing during the exercise.	Medium-high	5–10 min	Dynamically loosens deep calf muscle groups, increases intermuscular gliding and restores ankle range of motion
Type S - Hook Knife	Apply pressure sliding perpendicular to the muscle fibers over localized areas of stiffness and pain points in the calf.	low-middle	1 min	Deep and targeted relaxation of calf pain points.

### Control group

The control group in this study did not utilize equipment for ankle stabilization training and strength training of the peripheral muscle groups of the ankle joint. The specific training program employed by the control group was identical to that of the experimental group (refer to Table 2).

## Research measures

### Cumberland ankle instability tool

The Cumberland Ankle Instability Tool (CAIT) [43] evaluates the perceived degree of ankle instability in patients, encompassing the frequency, intensity, and impact of symptoms. The CAIT typically comprises a set of specific questions, each offering different scoring options. The total score typically ranges from 0 to 30 points.

### Foot and ankle ability measure

The Foot and Ankle Ability Measure (FAAM) is a scale utilized for evaluating ankle joint function [44]. It encompasses inquiries pertaining to pain, function, and quality of life, offering a comprehensive assessment of ankle instability in patients. The functional assessment comprises two levels: activities of daily living (FAAM-ADL) and sports activities (FAAM-SPORT). Scores typically range from 0 to 100 points, with 100 representing normal ankle joint function and 0 indicating severe limitations or complete inability to use the ankle joint.

### Ankle joint range of motion

In this study, a high-precision joint motion angle measurement device was utilized to evaluate the range of motion for various functions of the ankle joint in sitting and supine positions, encompassing measurements of ankle dorsiflexion and plantarflexion, as well as inversion and eversion of the foot [45–47].

**Fig. 2:**
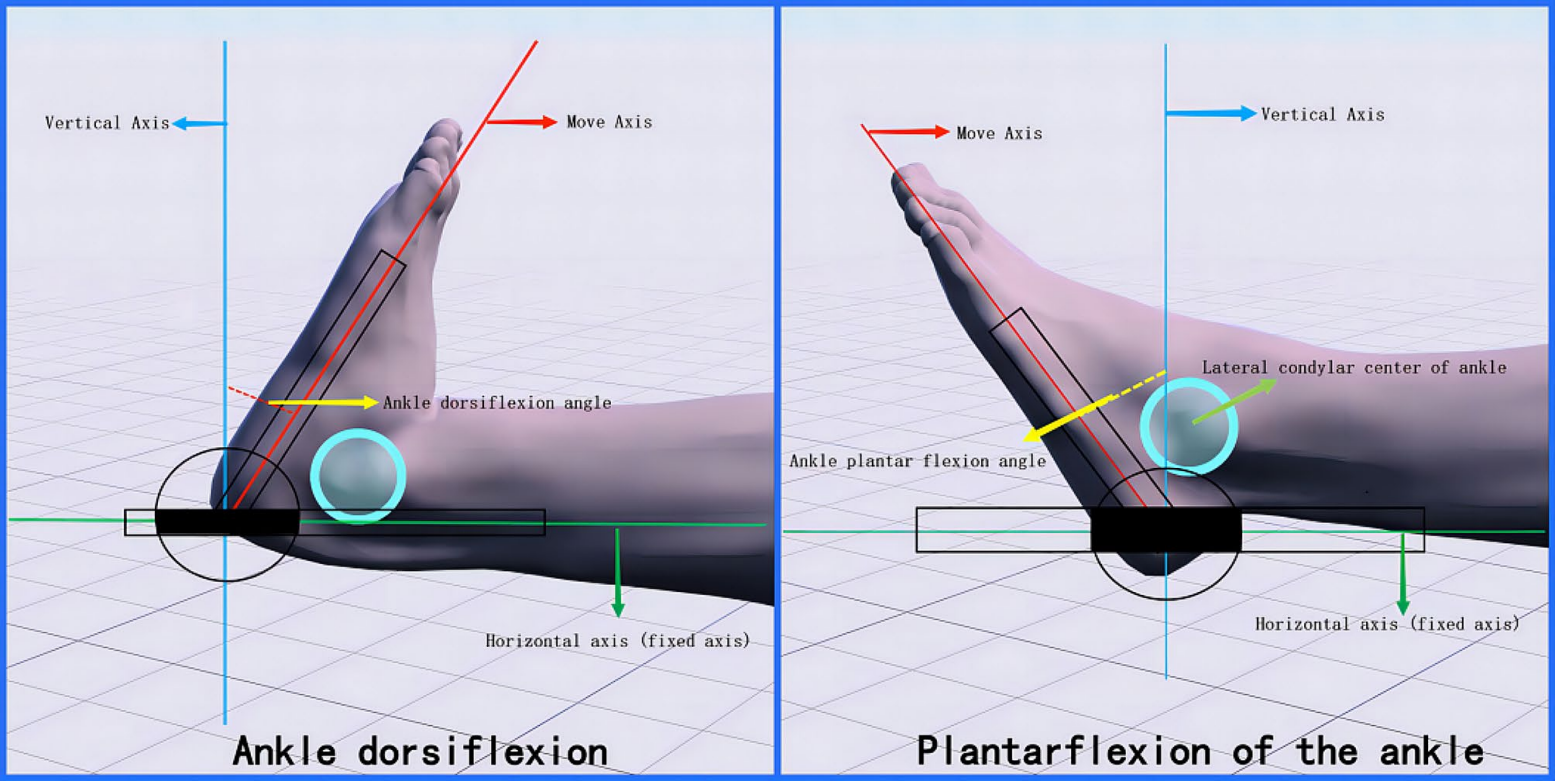
Range of motion measurements of dorsiflexion and plantarflexion of the foot

Measurement of ankle dorsiflexion and plantarflexion (refer to Fig. 2): The subject lies supine at the edge of the treatment table with the knee extended and the ankle joint in a neutral position. The center of the angle measurement device is aligned laterally with the subject’s ankle, using the fifth metatarsal as the longitudinal axis (axis of movement). The subject is instructed to execute ankle joint flexion by raising the foot from the neutral position or ankle joint extension by pushing the foot down, aiming to achieve the maximum range of motion with each movement until discomfort is felt or the maximum comfortable range is reached. The angle between the longitudinal axis and the neutral position horizontal axis represents the range of dorsiflexion or plantarflexion.

**Fig. 3:**
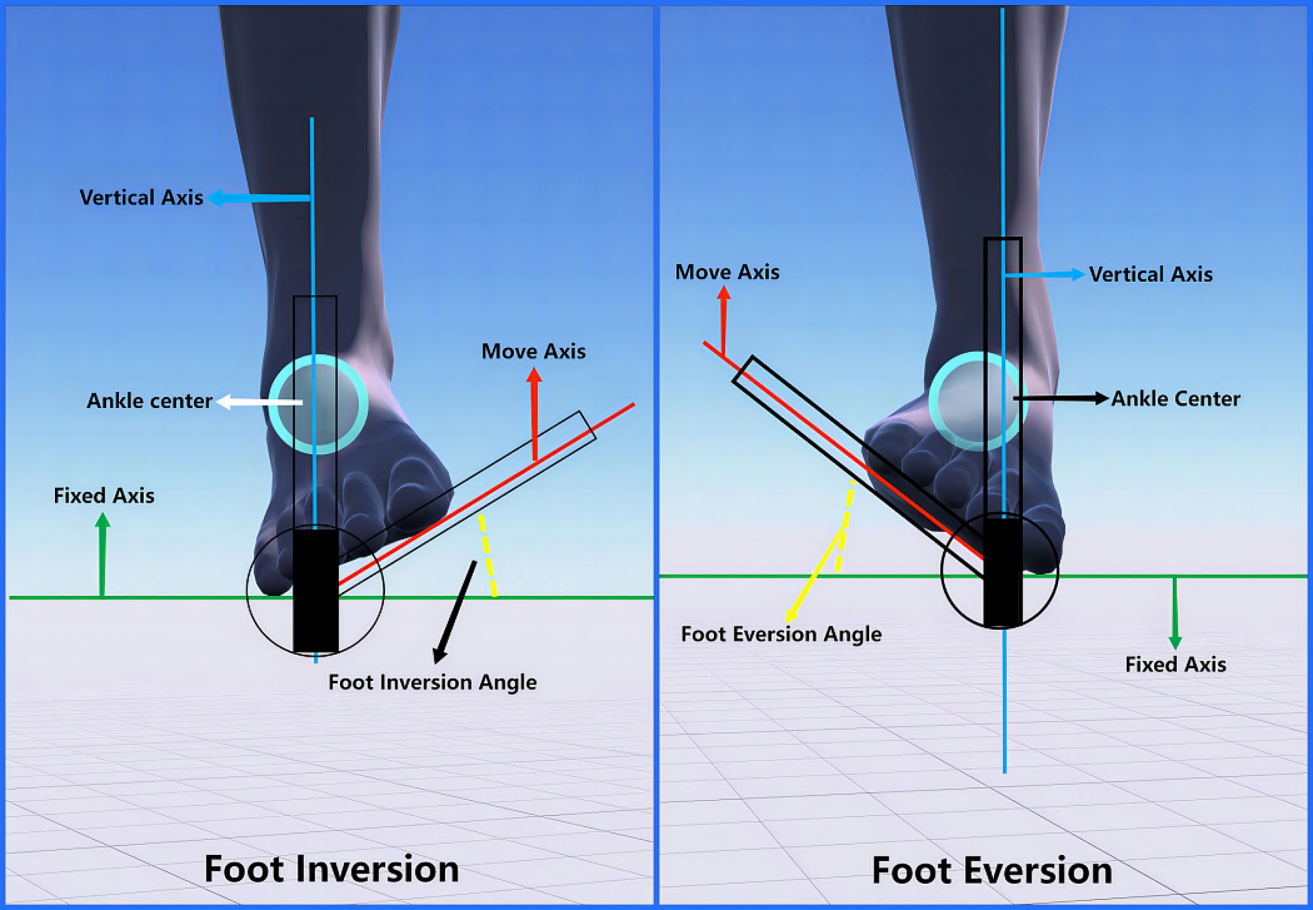
Range of motion measurements of foot valgus and valgus

Measurement of foot inversion and eversion (see Fig. 3): The subject is seated with the knee naturally flexed, and the ankle joint is in a neutral position. The fixed axis is the vertical axis of the sole of the foot, perpendicular to the longitudinal axis of the lower leg. The moving axis is the moving plantar surface of the foot, with the intersection of the two axes (fixed and moving) as the axis center. During measurement, the subject performs upward movement of the lateral edge of the foot (eversion) or downward movement of the lateral edge of the foot (inversion).

**Fig. 4:**
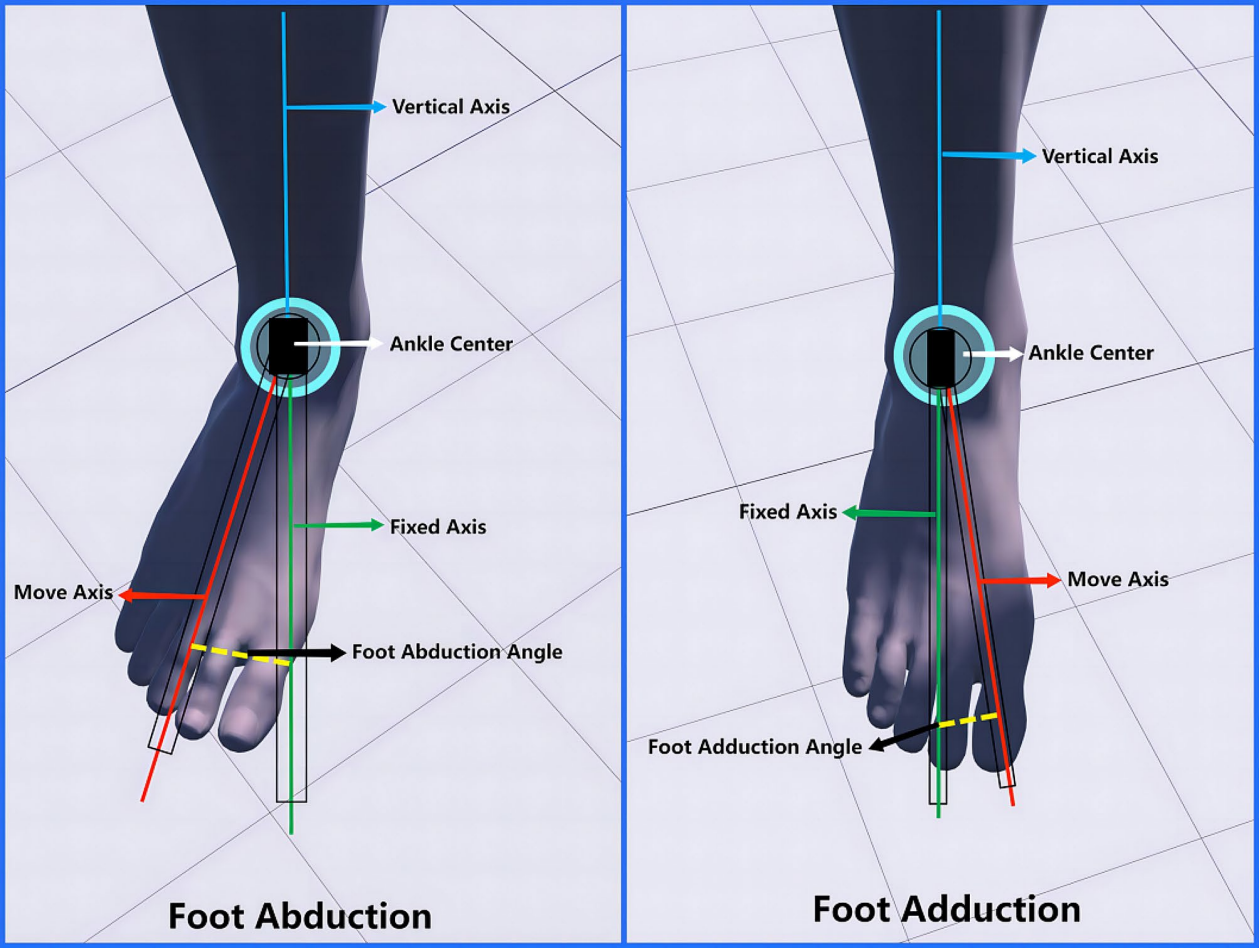
Range of motion measurements of foot abduction and adduction

Measurement of foot abduction and adduction (see Fig. 4): Position: The subject stands upright with the knee extended, ankle joint in a neutral position, and the lower leg fixed. The axis center is the midpoint of the anterior aspect of the ankle joint, medial malleolus, and lateral malleolus. The fixed axis is perpendicular to the longitudinal axis of the foot between the first and second metatarsals. The moving axis is the moving longitudinal axis of the foot. During measurement, the subject performs outward movement of the lateral edge of the foot (abduction) or inward movement of the medial edge of the foot (adduction).

### Ankle joint strength testing

The experimental assessment of strength data was carried out using a handheld digital muscle strength tester (model: FM-204 M series muscle strength tester). This muscle strength tester measures in units of Newtons (N), with a measurement range of ± 50kgf and a measurement accuracy of ± 0.5%FS (full scale) ± 1 digital peak value. It can measure both peak strength and instantaneous strength values. Therefore, in this study, a handheld digital muscle strength tester was utilized. The subjects assumed an appropriate position, with the base of the device secured to the ground, and force was applied to the ankle joint in various directions. The maximum strength in ankle dorsiflexion, plantarflexion, eversion, and inversion of the foot was recorded. Three repetitions of strength testing were conducted for each movement, and the average of the three maximum strength values was calculated.

### Statistical analysis

Data reading, testing, and statistical analysis were conducted using SPSS 26.0 statistical software in this study. Independent sample t-tests were employed for general subject information, as the continuous data followed a normal distribution. Given that ankle joint functional and range of motion indicators were measured at three time points (pre-intervention, initial intervention, and 4 weeks post-intervention), repeated measures analysis of variance (ANOVA) was utilized for statistical analysis and interpretation of the data. Ankle joint strength was assessed at two time points (pre-intervention and 4 weeks post-intervention), and paired t-tests were used for data analysis. A significance level of *P* < 0.05 was considered statistically significant.

For sample size calculation, this study utilized G.power 3.1.9.7, as the experiment involved three measurements on the distribution of two intervention groups. Based on the experiment’s characteristics, the final required sample size was 44/3 = 14.6 ≈ 15, and the number of subjects in this experiment was 30, aligning with the sample size calculation results.

## Results

### Baseline characteristics and recruitment results of participants

**Fig. 5:**
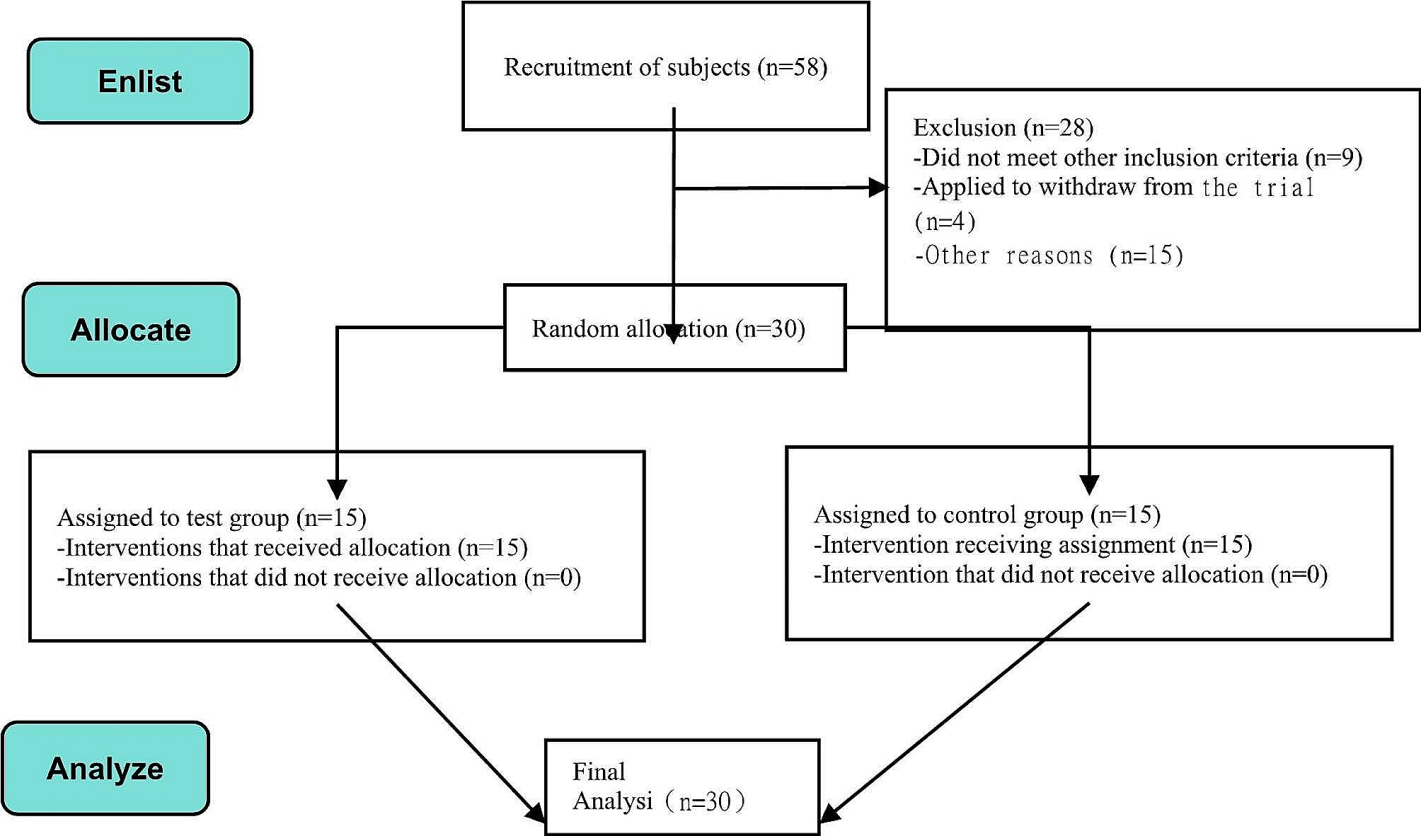
Flow chart of subject recruitment

A total of 58 individuals were initially recruited for this experiment. Following screening based on the inclusion and exclusion criteria, 30 specialized sports dance participants who met the experimental criteria were included (Refer to Fig. 5). These participants were randomly assigned to two groups: the Test group (*n* = 15, BFRT combined with IASTM) and the traditional ankle joint strength stability training group (*n* = 15). Detailed participant information comparison can be found in Table 4. The recruitment and allocation of participants, intervention measures, data recording, and statistical analysis for this experiment were all overseen by the first author. Basic participant information, including gender, age, height, weight, and duration of pain, was analyzed using paired t-tests, revealing no significant differences between the two groups (*p* > 0.05) (Refer to Table 4).

**Table 4:** Comparison of basic information of subjects in two groups

Variables	Test Group(*n* = 15)	Control Group (*n* = 15)	t	P
Age (n)	20.27 ± 1.79	19.60 ± 1.68	−0.19	0.47
Sex (m/f)	8/7	7/8		
Height (cm)	172.63 ± 9.13	174.40 ± 7.23	0.38	0.15
Weight (kg)	65.15 ± 23.55	60.27 ± 9.051	−0.46	0.07
Number of broken ankles in the past 1 year (n)	1.67 ± 0.64	2.00 ± 0.85	0.52	0.06
Affected ankle (n)				
Left side	11	10		
Right side	4	5		
Number of times whether the ankle was broken in training (n)	2.60 ± 1.12	2.13 ± 1.06	−0.13	0.63
Ankle sprains in the past 5 years (n)	2.73 ± 1.22	2.33 ± 1.11	0.22	0.41
yes	15	15		
No	0	0		
Time in specialization (years)	6.80 ± 3.69	7.13 ± 4.17	0.10	0.70

### CAIT score results

A repeated measures analysis of variance was performed on the CAIT scores at three different time points: pre-intervention, post-initial intervention, and 4 weeks post-intervention. The results indicated that the group factor did not yield a significant effect on CAIT scores (F = 0.23, *P* = 0.63). However, there was a noteworthy impact of measurement time on CAIT scores (F = 331.91, *P* < 0.05). Furthermore, the interaction between measurement time and group factor exhibited a significant effect on CAIT scores (F = 10.45, *P* < 0.05). These findings suggest that both the measurement time and the interaction between group and measurement time influenced the changes in CAIT scores among the participants. (Table 5).

**Table 5:** Multiple factor repeated measures ANOVA results and comparison of numerical changes with mean for the three time points of the CAIT scoring table

Results of Comparison of Changes in Test Values and Means at Three Time Points on the CAIT Rating Scale					CAIT Repeated Evaluation F-Test			
**Group**	**PRE**	**PTFI**	**P4WI**	**Comparison of multiple means**		***F***	***P***	***Bias η2***
	M ± SD	M ± SD	M ± SD		Group main effec	0.23	0.63	0.01
Test Group (*n* = 15)	9.53± 1.81	16.07± 2.46*&	26.27± 2.31*&**#**	PRE < PTFI < P4WI				
					Time point main effect	181.46	0.00	0.87
Control Group (*n* = 15)	10.93± 2.76	17.60± 4.58*&	22.37± 3.69*&**#**	PRE < PTFI < P4WI	Time point × group	9.17	0.00	0.25

*Note* represents a significant difference in the change in CAIT scores when compared to pre-intervention at within-group comparisons (*p* < 0.05); **&** represents a significant change when comparing within-groups after the first intervention and after the 4-week intervention (*p* < 0.05); **#** represents a significant change when comparing with between-groups (*p* < 0.05); M ± SD indicates mean ± standard deviation; PRE = Pre-Intervention; PTFI = Post The First Intervention; P4WI = Post 4 Weeks Of Intervention

When comparing between groups, a significant difference in CAIT scores was observed after 4 weeks of intervention (*P* > 0.05). The Test group exhibited higher CAIT scores than the control group, suggesting that the combined intervention had a more favorable therapeutic effect on ankle joint stability in the participants following the exercise treatment. When comparing within groups, both groups demonstrated significantly higher CAIT scores after the initial intervention and 4 weeks of intervention compared to pre-intervention (*P* < 0.05). Additionally, the CAIT scores after 4 weeks of intervention were also higher than those after the initial intervention (*P* < 0.05), indicating that both intervention groups had a notable impact on enhancing ankle joint stability in the participants. (Table 5).

### FAAM ankle functional assessment results

By conducting statistical analysis on the FAAM score sheets, including FAAM-ADL and FAAM-SPORT, related to ankle function:

### FAAM-adl score

The group factor did not yield a significant effect on FAAM-ADL scores (F = 2.63, *P* = 0.12). However, there was a notable impact of measurement time on FAAM-ADL scores (F = 128.428, *P* < 0.05). The interaction between measurement time and group factor did not yield a significant effect on FAAM-ADL scores (F = 2.63, *P* = 0.68). These findings suggest that the different groups and the interaction between group and measurement time did not influence FAAM-ADL scores, while different measurement times had a significant effect on FAAM-ADL scores.

When comparing between groups, no significant differences in FAAM-ADL scores were observed at the three time points (*P* > 0.05).

When comparing within groups, both groups exhibited significantly higher FAAM-ADL scores after the initial intervention and 4 weeks of intervention compared to pre-intervention (*P* < 0.05). Furthermore, the FAAM-ADL scores after 4 weeks of intervention were also higher than those after the initial intervention (*P* < 0.05), indicating that both intervention groups had a substantial positive impact on enhancing ankle joint function in daily activities for the participants. (Table 6).

**Table 6:** Multiple factor repeated measures ANOVA results and comparison of numerical changes with mean for the three time points of the FAAM-ADL scale

Results of Comparison of Changes in Test Values and Means at Three Time Points on the FAAM-ADL Rating Scale					FAAM-ADL Repeated Evaluation F-tests			
**Group**	**PRE**	**PTFI**	**P4WI**	**Comparison of multiple means**		***F***	***P***	***Bias η2***
	M ± SD	M ± SD	M ± SD		Group main effec	2.63	0.12	0.08
Test Group (*n* = 15)	41.67 ± 15.55	81.33±10.93*&	90.00±10.18*&	PRE < PTFI < P4WI				
					Time point main effect	128.42	0.00	0.82
Control Group (*n* = 15)	39.67±17.78	74.67±7.19*&	86.00±7.37*&	PRE < PTFI < P4WI	Time point × group	2.63	0.68	0.01

*Note* represents a significant difference in the change in FAAM-ADL scores when compared to pre-intervention at the time of within-group comparisons (*p* < 0.05); **&** represents a significant change when within-group comparisons were made after the first intervention and after the 4-week intervention (*p* < 0.05); M ± SD denotes mean ± standard deviation; PTFI = Post The First Intervention; P4WI = Post 4 Weeks Of Intervention

### FAAM-sport score

The group factor did not yield a significant effect on FAAM-SPORT scores (F = 0.22, *P* = 0.09). However, there was a noteworthy impact of measurement time on FAAM-SPORT scores (F = 273.84, *P* < 0.05). The interaction between measurement time and group factor did not yield a significant effect on FAAM-SPORT scores (F = 4.93, *P* = 0.02). These findings suggest that the different groups did not influence FAAM-SPORT scores, while different measurement times and the interaction between group and measurement time had a significant effect on FAAM-SPORT scores. (Specific data can be found in Table 7).

**Table 7:** Multiple factor repeated measures ANOVA results and comparison of numerical changes with mean for the three time points of the Foot range of motion(Inside-out angle、Outside-in angle)

Comparison of numerical changes in dorsiflexion and plantarflexion angles with the mean results							F-test for repeated evaluation of range of motion			
	**Test Group** (*n* = 15)			**Control Group** (*n* = 15)				***F***	***P***	***Bias η2***
	PRE	PTFI	P4WI	PRE	PTFI	P4WI				
	M ± SD	M ± SD	M ± SD	M ± SD	M ± SD	M ± SD				
**Inside-out angle**	29.40 ± 4.95	45.35 ± 6.54*#&	53.36 ± 8.00*#&	30.99 ± 4.07	34.84 ± 4.35*#&	38.31 ± 5.87*#&	Group main effec	17.92	0.00	0.39
							Time point main effect	182.20	0.00	0.86
							Time point × group	53.79	0.00	0.65
**Outside-in angle**	14.12 ± 2.49	17.87 ± 3.35*&	23.90 ± 3.48*&	15.44 ± 3.14	16.85 ± 3.10&	21.47 ± 3.68*&	Group main effec	0.42	0.51	0.01
							Group main effec	204.91	0.00	0.88
							Time point main effect	11.32	0.00	0.28

*Note* represents a significant difference in the change in dorsiflexion angle when compared to pre-intervention for within-group comparisons (*p* < 0.05); # represents a significant change in between-group comparisons with the Test group (*p* < 0.05); & represents a significant change in within-group comparisons after the first intervention and after the 4-week intervention (*p* < 0.05); M ± SD indicates mean ± standard deviation; PTFI = Post The First Intervention; P4WI = Post 4 Weeks Of Intervention

When comparing between groups, significant differences in FAAM-SPORT scores were observed after the initial intervention and 4 weeks of intervention (*P* < 0.05). The Test group exhibited significantly higher FAAM-SPORT scores than the control group, indicating that the combined intervention had a positive effect on enhancing daily physical activities for patients with chronic ankle instability.

When comparing within groups, both groups demonstrated significantly higher FAAM-SPORT scores after the initial intervention and 4 weeks of intervention compared to pre-intervention (*P* < 0.05). In the Test group, the FAAM-SPORT scores after 4 weeks of intervention were also higher than those after the initial intervention (*P* < 0.05). However, there was no significant difference in FAAM-SPORT scores between the initial intervention and 4 weeks of intervention in the control group (*P* > 0.05). (Table 8).

**Table 8:** Multiple factor repeated measures ANOVA results and comparison of numerical changes with mean for the three time points of the FAAM-SPORT scale

Results of Comparison of Changes in Test Values and Means at Three Time Points on the FAAM-SPORT Rating Scale					FAAM-SPORT Repeated Evaluation F-tests			
**Group**	**PRE**	**PTFI**	**P4WI**	**Comparison of multiple means**		***F***	***P***	***Bias η2***
	M ± SD	M ± SD	M ± SD		Group main effec	0.02	0.09	0.00
Test Group (*n* = 15)	32.3 ± 310.83	77.33±10.67*&**#**	93.33±4.88*&**#**	PRE < PTFI < P4WI				
					Time point main effect	273.84	0.00	0.91
Control Group (*n* = 15)	32.00±15.68	86.33±7.43*#	85.67±12.94*#	PRE < PTFI < P4WI	Time point × group	4.93	0.02	0.15

*Note* represents a significant difference in the change in FAAM-SPORT scores when compared to pre-intervention at within-group comparisons (*p* < 0.05); **&** represents a significant change when comparing within-groups after the first intervention and after the 4-week intervention (*p* < 0.05); **#** represents a significant change when comparing with between-groups (*p* < 0.05); M ± SD indicates mean ± standard deviation; PTFI = Post The First Intervention; P4WI = Post 4 Weeks Of Intervention

### Measurement results of ankle joint range of motion

In this experiment, a high-precision joint motion angle measurement device was used to measure the range of motion for different functions of the ankle joint in sitting and supine positions, including measurements of ankle dorsiflexion and plantarflexion angles, inversion and eversion angles of the foot, and abduction and adduction angles of the foot.

### Dorsiflexion and plantarflexion

In the assessment of ankle dorsiflexion and plantarflexion angles in the supine position, the results of repeated measures analysis of variance conducted at three different time points (pre-intervention, post-initial intervention, and 4 weeks post-intervention) revealed the following: The group factor had a significant effect on ankle dorsiflexion angle (F = 17.69, *P* < 0.05), but not on ankle plantarflexion angle (F = 1.79, *P* = 0.19). Measurement time exhibited a significant effect on both ankle dorsiflexion and plantarflexion angles (F = 1271.87, *P* < 0.05; F = 286.75, *P* < 0.01). Additionally, the interaction between measurement time and group factor had a significant effect on ankle dorsiflexion and plantarflexion angles (F = 147.76, *P* < 0.01; F = 24.90, *P* < 0.01). These findings indicate that different groups, measurement times, and the interaction between group and measurement time had a significant effect on ankle dorsiflexion and plantarflexion angles.

When comparing between groups, significant differences in ankle dorsiflexion angles were observed after the initial intervention and 4 weeks of intervention (*P* < 0.05), while ankle plantarflexion angles exhibited significant differences only after the initial intervention (*P* < 0.05).

When comparing within groups, both groups demonstrated significantly higher ankle dorsiflexion and plantarflexion angles after the initial intervention and 4 weeks of intervention compared to pre-intervention (*P* < 0.05). Furthermore, after 4 weeks of intervention, both the Test group and the control group exhibited higher ankle dorsiflexion and plantarflexion angles compared to after the initial intervention (*P* < 0.05). (Table 9).

**Table 9:** Multiple factor repeated measures ANOVA results and comparison of numerical changes with mean for the three time points of the Foot range of motion(Dorsiflexion angle、Plantarflexion angle)

Comparison of numerical changes in dorsiflexion and plantarflexion angles with the mean results							F-test for repeated evaluation of range of motion			
	**Test Group** (*n* = 15)			**Control Group** (*n* = 15)				***F***	***P***	***Bias η2***
	PRE	PTFI	P4WI	PRE	PTFI	P4WI				
	M ± SD	M ± SD	M ± SD	M ± SD	M ± SD	M ± SD				
**Dorsiflexion angle**	15.40 ± 2.49	23.65 ± 2.74*#&	37.26 ± 3.19*#&	16.72 ± 3.14	19.32 ± 3.14*#&	27.32 ± 3.16*#&	Group main effec	17.69	0.00	0.38
							Time point main effect	1271.87	0.00	0.97
							Time point × group	147.76	0.00	0.84
**Plantarflexion angle**	38.06 ± 6.83	50.97 ± 6.37*#&	61.27 ± 8.20*&	41.95 ± 3.11	44.01 ± 3.69**#**&	56.08 ± 3.92*&	Group main effec	1.79	0.19	0.06
							Group main effec	286.75	0.00	0.91
							Time point main effect	24.90	0.00	0.47

*Note* represents a significant difference in the change in dorsiflexion angle when compared to pre-intervention for within-group comparisons (*p* < 0.05); **#** represents a significant change in between-group comparisons with the Test group (*p* < 0.05); **&** represents a significant change in within-group comparisons after the first intervention and after the 4-week intervention (*p* < 0.05); M ± SD indicates mean ± standard deviation; PTFI = Post The First Intervention; P4WI = Post 4 Weeks Of Intervention

### Inversion and eversion

In the assessment of ankle inversion and eversion angles in the sitting position, the results of repeated measures analysis of variance conducted at three different time points (pre-intervention, post-initial intervention, and 4 weeks post-intervention) revealed the following: The group factor had a significant effect on ankle inversion angle (F = 17.92, *P* < 0.05), but not on ankle eversion angle (F = 0.42, *P* > 0.51). Measurement time exhibited a significant effect on both ankle inversion and eversion angles (F = 182.20, *P* < 0.05; F = 204.91, *P* < 0.05). Additionally, the interaction between measurement time and group factor had a significant effect on ankle inversion and eversion angles (F = 53.79, *P* < 0.05; F = 11.32, *P* < 0.05). These findings indicate that different groups, measurement times, and the interaction between group and measurement time had a significant effect on ankle inversion angle measurements.

When comparing between groups, significant differences in ankle inversion angles were observed after the initial intervention and 4 weeks of intervention (*P* < 0.05), with greater increases observed in the Test group. However, there were no significant differences in ankle eversion angles between the two intervention groups (*P* > 0.05), indicating that both intervention groups had no significant effect on ankle eversion angles in CAI patients.

When comparing within groups, both groups demonstrated significantly higher ankle inversion angles after the initial intervention and 4 weeks of intervention compared to pre-intervention (*P* < 0.05). Furthermore, there was a significant difference in ankle inversion angles between 4 weeks of intervention and the initial intervention within each group, with the best improvement observed after 4 weeks of intervention. For ankle eversion angles, the Test group exhibited significant increases after the initial intervention and 4 weeks of intervention compared to pre-intervention (*P* < 0.05). Additionally, ankle eversion angles after 4 weeks of intervention were significantly higher than those after the initial intervention and pre-intervention (*P* < 0.05) in the Test group. However, in the control group, ankle eversion angles were only significantly higher after 4 weeks of intervention compared to after the initial intervention and pre-intervention (*P* < 0.05). (Table 7)

### Abduction and adduction

In the assessment of ankle abduction and adduction angles in the sitting position, the results of repeated measures analysis of variance conducted at three different time points (pre-intervention, post-initial intervention, and 4 weeks post-intervention) revealed the following: The group factor did not yield a significant effect on ankle adduction angles (F = 0.06, *P* = 0.80), but did have a significant effect on ankle abduction angles (F = 13.09, *P* < 0.05). Measurement time exhibited a significant effect on both ankle abduction and adduction angles (F = 94.01, *P* < 0.05; F = 312.68, *P* < 0.05). Additionally, the interaction between measurement time and group factor did not yield a significant effect on ankle adduction angles (F = 2.79, *P* = 0.08), but did have a significant effect on ankle abduction angles (F = 9.01, *P* < 0.05). These findings indicate that different groups, measurement times, and the interaction between group and measurement time had a significant effect on ankle abduction angles, but not on ankle adduction angles.

When comparing between groups, significant differences in ankle abduction angles were observed after the initial intervention and 4 weeks of intervention (*P* < 0.05), with greater increases observed in the Test group. However, there were no significant differences in ankle adduction angles between the two intervention groups (*P* > 0.05), indicating that both intervention groups had no significant effect on ankle adduction angles in CAI patients.

When comparing within groups, both groups demonstrated significantly higher ankle abduction and adduction angles after the initial intervention and 4 weeks of intervention compared to pre-intervention (*P* < 0.05). Furthermore, there were significant differences in ankle abduction angles between 4 weeks of intervention and the initial intervention within each group, with the best improvement observed after 4 weeks of intervention. For ankle adduction angles, there were no significant differences between the two time points (*P* > 0.05) in both groups.( Table 10.

**Table 10:** Multiple factor repeated measures ANOVA results and comparison of numerical changes with mean for the three time points of the Foot range of motion(Inward Angle、Outward Angle)

Comparison of numerical changes in dorsiflexion and plantarflexion angles with the mean results							F-test for repeated evaluation of range of motion			
	**Test Group** (*n* = 15)			**Control Group** (*n* = 15)				***F***	***P***	***Bias η2***
	PRE	PTFI	P4WI	PRE	PTFI	P4WI				
	M ± SD	M ± SD	M ± SD	M ± SD	M ± SD	M ± SD				
**Inward Angle**	25.17 ± 2.49	31.87 ± 3.96*&	37.75 ± 4.88*&	26.38 ± 3.22	32.10 ± 4.66*&	35.35 ± 5.63*&	Group main effec	0.06	0.80	0.00
							Time point main effect	94.01	0.00	0.77
							Time point × group	2.79	0.08	0.09
**Outward Angle**	20.82 ± 2.50	49.22 ± 4.51*#	48.78 ± 6.00*#	22.14 ± 3.15	41.52 ± 5.39*#	43.09 ± 5.05*#	Group main effec	13.09	0.00	0.33
							Group main effec	312.68	0.00	0.91
							Time point main effect	9.01	0.00	0.24

*Note* represents a significant difference in the change in dorsiflexion angle when compared to pre-intervention for within-group comparisons (*p* < 0.05); & represents a significant change in within-group comparisons after the first intervention and after the 4-week intervention (*p* < 0.05); # represents a significant change in between-group comparisons with the Test group (*p* < 0.05); M ± SD indicates mean ± standard deviation; PTFI = Post The First Intervention; P4WI = Post 4 Weeks Of Intervention

### Ankle joint strength testing

In this experiment, a handheld muscle strength tester was utilized to measure the maximum strength of various functions (ankle dorsiflexion, ankle plantarflexion, ankle inversion, and ankle eversion) in CAI patients.

Within-group comparisons demonstrated significant improvements in ankle strength for all four movement patterns before and after the intervention in both intervention groups (*P* < 0.05). These findings indicate that both interventions effectively enhanced ankle joint strength in CAI patients.

Between-group comparisons revealed that the Test group exhibited significantly higher ankle plantarflexion and ankle inversion strength after the intervention compared to the control group (*P* < 0.05). However, there were no significant differences between the groups in other ankle strength measurements (*P* > 0.05). ( Table 11).

**Table 11:** Comparison of mean values of different functional strengths of the ankle joint in standing position

Group	Projects/Strengths	Pre-Intervention/N	Post 4 Weeks Of Intervention/N	Comparison of multiple means
		M ± SD	M ± SD	
Test Group (*n* = 15)	Dorsiflexion	41.40 ± 18.06*	123.53 ± 25.45*	PRE < P4WI
	Plantar Flexion	90.72 ± 16.88*	146.08 ± 25.32*a	PRE < P4WI
	Adduction	33.51 ± 10.36*	64.61 ± 12.43*a	PRE < P4WI
	Abduction	48.66 ± 12.62*	78.12 ± 17.67*	PRE < P4WI
Control Group (*n* = 15)	Dorsiflexion	49.79 ± 16.37*	123.86 ± 31.33*	PRE < P4WI
	Plantar Flexion	83.74 ± 18.92*	125.610 ± 28.39*a	PRE < P4WI
	Adduction	30.41 ± 11.33*	50.90 ± 13.60*a	PPRE < P4WI
	Abduction	50.40 ± 12.02*	70.57 ± 16.82*	PRE < P4WI

*Note* represents a significant difference in the change in mean strength values before and after the intervention for within-group comparisons (*p* < 0.05); **a** represents a significant difference in the change in mean strength values for between-group comparisons (*p* < 0.05)

## Discussion

This study showcased the notable therapeutic effects of blood flow restriction training combined with IASTM on Chronic Ankle Instability (CAI) patients engaged in sports dance. Analysis of the four-week intervention results revealed significant enhancements in ankle joint stability for both intervention groups, as evidenced by substantial changes in CAIT scores. However, the experimental group exhibited superior improvements in stability compared to the control group. Both groups significantly bolstered ankle joint function in terms of daily activities and sports function, with no significant differences observed in FAAM-ADL scores between the groups. Nevertheless, the experimental group achieved significantly higher FAAM-SPORT scores than the control group, indicating a more robust recovery of sports function with the intervention. Both groups demonstrated varying degrees of improvement in ankle joint range of motion, particularly in ankle dorsiflexion, ankle eversion, and ankle inversion, which significantly improved after the initial intervention and 4 weeks of intervention. The experimental group yielded better treatment outcomes than the control group. However, there were no significant improvements in ankle adduction and ankle abduction. In terms of ankle strength improvement, both intervention groups exhibited increased ankle strength for all four movement patterns (dorsiflexion, plantarflexion, inversion, and eversion) before and after the intervention. Furthermore, the experimental group displayed significantly higher ankle plantarflexion and ankle inversion strength than the control group.Based on the aforementioned research findings, it is evident that both intervention approaches partially alleviate symptoms in athletes with chronic ankle instability (CAI). Both the BFRT combined with IASTM intervention group and the traditional ankle strength training group significantly enhanced stability, functionality, strength, and range of motion in CAI patients. Moreover, the combined intervention in the experimental group exhibited superior efficacy in ankle stability, daily functional movement, dorsiflexion, and eversion range of motion compared to the control group.

The pathological mechanisms of Chronic Ankle Instability (CAI) encompass alterations at multiple levels. Primarily, CAI often involves chronic damage to the lateral ligaments, particularly those of the fibula [4]. In the case of sports dance athletes, recurrent ankle sprains or inadequate rehabilitation can lead to gradual ligament laxity, resulting in diminished effective support for the ankle joint. Consequently, this can lead to excessive joint displacement during regular movement, thereby heightening joint instability [4]. This factor significantly contributes to the frequent occurrence of ankle injuries in sports dance athletes. Furthermore, habitual sprains may induce sensory nerve abnormalities, resulting in reduced perception of joint position and a substantial increase in the risk of re-injury. Additionally, prolonged ligament damage can also lead to impairments in motor control, encompassing muscle coordination and balance issues. These functional impairments can culminate in the loss of precise control over the ankle joint during daily activities and sports, further elevating the risk of injury [9]. Chronic ligament damage and laxity contribute to reduced overall ankle joint stability, rendering the joint more susceptible to abnormal displacement during regular movement and loading. Moreover, damaged ligaments may prompt adaptive changes in the surrounding soft tissues, including muscle atrophy and morphological alterations in tendons. These adaptive changes further compromise the structural support for the ankle joint and create conducive conditions for additional functional impairments [10]. Consequently, this study aims to alleviate symptoms in CAI patients by leveraging the distinctive mechanisms of BFRT combined with IASTM at various levels.

The mechanisms underlying the effects of Blood Flow Restriction Training (BFRT) on muscle growth primarily involve metabolic and mechanical tension aspects. By utilizing a loose pneumatic cuff at the base of the limb to restrict blood flow, a hypoxic environment is created, leading to increased lactate accumulation in the muscles [48]. This physiological state triggers a series of metabolic reactions, including the release of growth hormones, which aid in promoting muscle growth factors. Additionally, BFRT restricts blood flow and reduces oxygen supply to the muscles, resulting in increased accumulation of metabolites such as lactate, which stimulates the neuromuscular system and enhances mechanical tension in the muscles, leading to increased muscle strength and volume at lower loads [49]. Therefore, in this experiment, BFRT is employed to restrict blood flow and reduce oxygen supply to the lower limb muscle groups, stimulating the neuromuscular system of the surrounding muscles of the ankle joint, enhancing muscle strength, and achieving high-intensity training at lower loads. This not only promotes the stability of the ankle joint by strengthening the muscle groups involved but also improves neuromuscular control, ultimately enhancing ankle joint stability.

Instrument-Assisted Soft Tissue Mobilization (IASTM), widely used in the field of physical therapy, can enhance treatment effects when combined with other physical therapy modalities such as hot/cold packs and electrical stimulation [25, 26]. In the field of rehabilitation medicine, IASTM is extensively employed for treating various types of sports injuries, including muscle strains and ligament damage. In sports medicine, IASTM techniques can assist athletes in rapid recovery and improve athletic performance [27]. During the treatment process, therapists utilize IASTM tools to perform scraping on the patient’s soft tissues, aiming to release adhesions and promote blood circulation [28]. Additionally, therapists adjust the pressure and intensity of scraping based on patient feedback to ensure treatment comfort and effectiveness. IASTM not only helps prevent and treat sports injuries, enabling athletes to maintain a healthy physical state, but also demonstrates significant potential applications in the fields of rehabilitation medicine, sports medicine, and orthopedic surgery. Stanek et al. [50] conducted a study demonstrating that Instrument-Assisted Soft Tissue Mobilization (IASTM) significantly improves restricted ankle joint flexion and enhances the range of motion of the ankle joint. Moreover, a substantial body of research has shown that IASTM can enhance short-term joint mobility and alleviate patient pain, making it a commendable physical therapy modality in clinical treatment [31, 51, 52].In summary, the use of Instrument-Assisted Soft Tissue Mobilization (IASTM) to address symptoms of Chronic Ankle Instability (CAI) is highly suitable as it not only targets ankle joint restrictions but also stimulates soft tissue surfaces, triggers local inflammatory responses, promotes blood circulation, and regulates neural functions, effectively alleviating pain, improving movement impairments, and facilitating the rehabilitation process. This study found that the combination of Blood Flow Restriction Training (BFRT) and IASTM indeed yields favorable therapeutic effects for CAI patients in sports dance. However, this study has limitations such as a small sample size, short treatment intervention period, incomplete and subjective quantitative measures, and a predominantly athlete population. Future research should consider expanding the sample size, including diverse populations, and incorporating more objective measures to enhance the reliability of the combined therapy’s effectiveness.

## Data Availability

The data underlying this paper, which includes the privacy of the individuals involved, cannot be made public for the following reasons. These data will be shared with the respective authors upon reasonable request.If you need the data, you can contact me via my email.784389072@qq.com.
